# Isolation and Optimization of a Broad-Spectrum Synthetic Antimicrobial Peptide, Ap920-WI, from *Arthrobacter* sp. H5 for the Biological Control of Plant Diseases

**DOI:** 10.3390/ijms241310598

**Published:** 2023-06-25

**Authors:** Li Zhao, Md. Samiul Islam, Pei Song, Li Zhu, Wubei Dong

**Affiliations:** Department of Plant Pathology, College of Plant Science and Technology and the Key Lab of Crop Disease, Monitoring & Safety Control in Hubei Province, Huazhong Agricultural University, Wuhan 430070, China; zhao_li@webmail.hzau.edu.cn (L.Z.); samiulislam@webmail.hzau.edu.cn (M.S.I.); songpei@webmail.hzau.edu.cn (P.S.); zl_888@webmail.hzau.edu.cn (L.Z.)

**Keywords:** *Bacillus subtilis* expression system, genome library, antimicrobial peptides (AMPs), synthetic antimicrobial peptide (SAMP), broad-spectrum activity

## Abstract

Antimicrobial peptides (AMPs) are naturally occurring molecules found in various organisms that can help to defend against invading microorganisms and reduce the likelihood of drug resistance development. This study focused on the isolation of new AMPs from the genome library of a Gram-positive bacterium called *Arthrobacter* sp. H5. To achieve this, we used the *Bacillus subtilis* expression system and employed bioinformatics techniques to optimize and modify the peptides, resulting in the development of a new synthetic antimicrobial peptide (SAMP). Ap920 is expected to be a new antimicrobial peptide with a high positive charge (+12.5). Through optimization, a new synthetic antimicrobial peptide, Ap920-WI, containing only 15 amino acids, was created. Thereafter, the antimicrobial and antifungal activities of Ap920-WI were determined using minimum inhibitory concentration (MIC) and the concentration for 50% of maximal effect (EC_50_). The Ap920-WI peptide was observed to target the outer membrane of fungal hyphae, leading to inhibition of growth in *Rhizoctonia Solani*, *Sclerotinia sclerotiorum*, and *Botrytis cinerea*. In plants, Ap920-WI showed significant antifungal activity and inhibited the infestation of *S. sclerotiorum* on rape leaves. Importantly, Ap920-WI was found to be safe for mammalian cells since it did not show any hemolytic activity against sheep red blood cells. Overall, the study found that the new synthetic antimicrobial peptide Ap920-WI exhibits broad-spectrum activity against microorganisms and may offer a new solution for controlling plant diseases, as well as hold potential for drug development.

## 1. Introduction

The increasing prevalence of multidrug-resistant bacterial infections due to overuse of antibiotics is posing significant challenges to crop production and human health care [1]. To combat this problem, identifying new antimicrobial genes is crucial. Antimicrobial peptides (AMPs) are an important component of organisms that inhibit harmful microorganisms by disrupting their cell membranes [2]. AMPs have hydrophilic and hydrophobic properties [3], positive charge, a low number of amino acids and helical motifs [4], and broad-spectrum antibacterial, antiviral, antitumor, immunomodulatory, and wound-healing activities [5,6], making them a promising candidate for developing new antibiotic drugs. AMPs can be classified as natural antimicrobial peptides (NAMPs) or synthetic antimicrobial peptides (SAMPs). NAMPs are small-molecule polypeptides encoded by genes that are present in organisms [7,8] with broad-spectrum antimicrobial activity [9]. However, directly isolating NAMPs from natural sources can be expensive, time-consuming, and environmentally unfavorable, especially when peptides are discovered in rare animals with small populations [10]. Alternatively, SAMPs are produced by cloning foreign genes onto specialized vectors and expressing them in host cells using recombinant DNA technologies, such as the bacterial expression system, which is the most efficient method in recent times [10,11].

In recent years, the development of SAMPs has attracted much attention due to their potential use in biological control of plant diseases. They can be used to obtain small-molecule polypeptides with high antibacterial activity, low toxicity, and high stability, which can be used to control some economically important phytosanitary disease pathogens [12]. Additionally, synthetic antimicrobial peptide genes can also be transferred into plants for expression, thereby enhancing plants’ disease resistance [13]. For example, synthetic peptide D4E1 can be transferred to cotton for expression to enhance its disease resistance to *Aspergillus flavus* [14].

Our laboratory has recently developed an efficient method for screening new antimicrobial potentials using the *Bacillus subtilis* expression system, which enables the purification of potential SAMPs [15,16,17,18]. *B. subtilis* is a Gram-positive soil bacterium [19] that is widely used due to its clear background of genetic research [20], no significant codon preference and therefore no optimization of DNA sequences required for the expression of exogenous proteins, absence of endotoxin production [21,22], rapid growth, and suitability for large-scale fermentation [23]. In addition, *B. subtilis* is non-pathogenic and can secrete recombinant proteins into the culture media using various signal peptides [24], making it a popular choice for producing heterologous proteins, enzymes, and other substances [25]. However, in silico tools are also now popular as an efficient method for large-scale screening and detection of antimicrobial peptides (AMPs) [26]. For example, CAMP_R4_ predicts AMP using machine learning algorithms such as random forest (RF), discriminant analysis (DA), and support vector machines (SVM) [27]. APD3 provides detailed peptide information, making it a helpful tool for scanning, customizing, and estimating AMPs [27]. Furthermore, peptide-based prediction and classification tools such as dbAMP, ClassAMP, iAMPpred, and AntiBp are used to predict the function and type of AMPs as antifungal, antibacterial, or antiviral agents [28].

In this study, our aim is to isolate, identify, and optimize the new SAMP Ap920-WI from *Arthrobacter* sp. H5 using in vitro and in silico analyses and examine its antimicrobial activity and mode of application against several crucial phytopathogens.

## 2. Results

### 2.1. Screening of Antibacterial Genes from Arthrobacter *sp.* H5 Genome Library and Verification of Their Activity

A genome library of Arthrobacter sp. H5 was constructed using the *B. subtilis* expression system (Figure S1), resulting in a total of 3350 transformants obtained. The quality of the inserted fragment was determined by PCR, revealing that most of the detected bands were above 500 bp (Figure S2), indicating that genomic DNA fragments of different sizes were successfully inserted.

**Figure 1:**
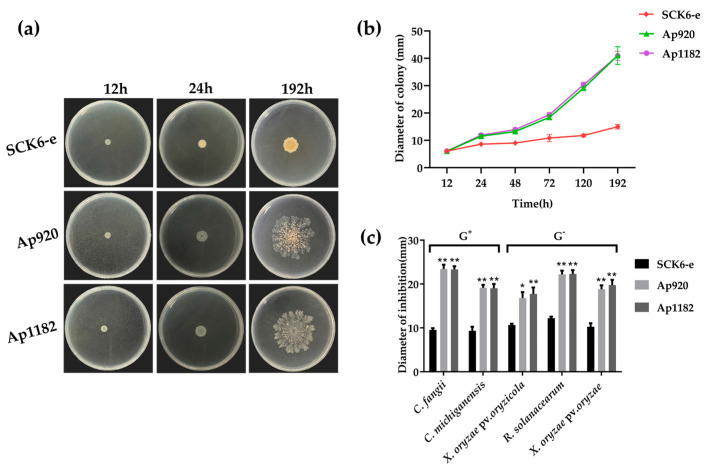
The effect and antibacterial activity of *Ap920* and *Ap1182* genes on the host cell *B. subtilis* were evaluated. The engineered *B. subtilis* bacteria strains harboring empty vector, *Ap920*, and *Ap1182* genes were individually placed on Lysogenic Broth (LB, 10 mg/L Kan) agar medium plates and incubated in a 37 °C incubator. The strains were regularly observed and photographed, and the growth diameter was recorded. (**a**) The SCK6-e strain, Ap920 strain, and Ap1182 strain were cultured for 12 h, 24 h, and 192 h; (**b**) data analysis of the strain’s growth diameter; (**c**) extracellular crude protein of Ap920 and Ap1182 strains were compared with SCK6-e, and their effects on the target bacteria’s bacteriostatic activity of Gram-positive (G+) and Gram-negative bacteria (G− were evaluated. The data were collected from three independent experiments: the vertical bars represent the standard deviation (SD); *T*-tests were conducted to evaluate the significance levels at * *p* < 0.05 and ** *p* < 0.01.

Transformant strains that caused damage to host cells were screened out. Compared with the control *B. subtilis* SCK6-e, the engineered *B. subtilis* bacteria with exogenous gene insertion showed autolysis and escape, leading to complete lysis of the host strain. The Ap920 strain showed autolysis after 24 h of growth, and after 192 h of growth, the strain was completely lysed with some bacteria remaining, and the contents diffused outwards, demonstrating an escape phenomenon. On the other hand, the Ap1182 strain did not exhibit autolysis after 24 h of growth, but after 192 h of growth, the strain was completely lysed with only a small amount of bacteria remaining and the contents overflowing (Figure 1a). Observing the growth diameter of the strains, the escape speeds of the Ap920 and Ap1182 strains were roughly the same (Figure 1b), but the mechanism of lysing the bacteria differed, which may be related to their antibacterial mode of action. These results suggested that the Ap920 and Ap1182 genes may be antibacterial genes, and the lysed content of the strain may contain gene expression products. Consequently, these two strains were selected for antibacterial activity verification (Table S1).

To verify the antibacterial activity of engineered *B. subtilis* bacteria Ap920 and Ap1182, extracellular crude protein was extracted using the saturated ammonium sulfate precipitation method, and the antibacterial activity was verified. The results showed that (Figure 1c), compared with the control SCK6-e, the extracellular crude protein of the Ap920 and Ap1182 strains had a significant effect on Gram-positive bacteria (*Clavibacter. fangii* and *Clavibacter. michiganensis*) and Gram-negative bacteria (*Xanthomonas. oryzae* pv. *oryzicola*, *Rastonia. Solanacearum*, and *Xanthomonas. oryzae* pv. *oryzae*), exhibiting significant antibacterial activity. Therefore, the extracellular crude protein of Ap920 and Ap1182 strains can be purified to obtain pure antimicrobial peptides Ap920 and Ap1182 for the next experiment.

### 2.2. Purification of Antimicrobial Peptides Ap920 and Ap1182, Bioinformatics Analysis and Determination of Minimum Inhibitory Concentration (MIC) Value

**Figure 2:**
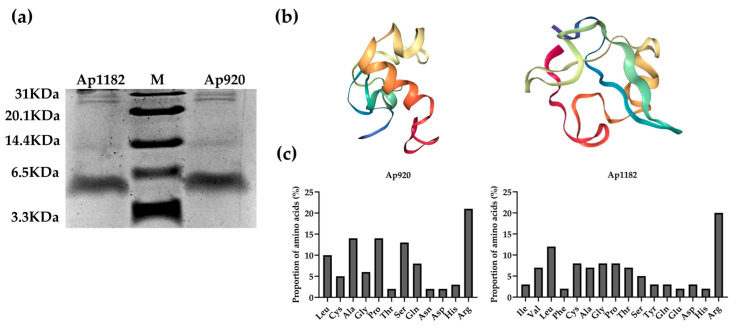
Tricine-SDS-PAGE electrophoresis and bioinformatics analysis of Ap920 and Ap1182. The samples of pure antimicrobial peptides Ap920 and Ap1182 were purified by Ni column, and the expression of the samples was detected by Tricine-SDS-PAGE and their sizes were verified. (**a**) Tricine-SDS-PAGE electrophoresis image: lane M is the pre-stained ultra-low molecular weight marker. (**b**) The 3D structures of the antimicrobial peptides Ap920 and Ap1182 were predicted according to C-I-TASSER of The Zhang Lab, and the 3D structures of the antimicrobial peptides were visualized using the NGL viewer 2.0.0 (http://nglviewer.org/ngl/, accessed on 28 February 2022). (**c**) According to the prediction results of the APD3, the amino acid composition of the antimicrobial peptides Ap920 and Ap1182 was analyzed, and the data were visualized using GraphPad Prism 9.

We examined whether the purified antimicrobial peptides Ap920 and Ap1182 were expressed by Tricine-SDS-PAGE, and the molecular weights of Ap920 (6.8 kDa) and Ap1182 (6.3 kDa) were verified. The results showed that the molecular weights of Ap920 and Ap1182 expressed were consistent with the predicted weights (Figure 2a). Next, we measured the minimum inhibitory concentration (MIC) value of Ap920 and Ap1182 against the five indicator pathogenic bacteria using the gradient dilution method. The results showed (Table 1) that their MIC values ranged from 50–140 μg/mL.

Based on the prediction information of APD3 and Expasy, the amino acid composition of Ap1182 is more complex than that of Ap920 and has the highest percentage of hydrophobic amino acids in the composition (Figure 2c). The number of positive charges, Wimley–White whole-residue hydrophobicity, molecular weight, protein-binding potential, and PI values of Ap920 are larger than those of Ap1182, but its hydrophobicity and GRAVY values are smaller (Table S2). In the 3D structure predicted by The Zhang Lab (Figure 2b), the structure of Ap920 is dominated by α-helix and there is an obvious α-helix, while the structure of Ap1182 is dominated by irregular coils and the helical structure is not obvious. Additionally, in the BLAST analysis of NCBI, we did not find any sequence corresponding to Ap920 and Ap1182 (Table S1).

Amino acid composition and the number of positive charges are the key factors affecting the antibacterial activity of antimicrobial peptides. The prediction analysis results show that the antimicrobial peptide Ap920, which has a simpler amino acid composition and a larger number of positive charges, exhibits better antimicrobial peptide properties. According to the experimental verification, we found that the antimicrobial peptide Ap920 has good antimicrobial activity. In order to verify whether the active region of Ap920 still has antimicrobial activity, we predicted the active region of Ap920 by bioinformatics, optimized and modified Ap920 to obtain a new antimicrobial peptide, and then verified its effect by the same experimental method.

### 2.3. Optimization and Transformation of Ap920 Using Bioinformatics and Antimicrobial Peptide Properties

We utilized three algorithms (SVM, ANN, and QM) of the AntiBP serve website to predict the most suitable N-terminus and C-terminus sequence peptides. Then, we used three algorithms (SVM, RF, and DA) of the CAMP_R4_ website to predict the probability of each site’s amino acids to becoming antimicrobial peptides. Finally, we mutated and deleted amino acids based on their properties to obtain the optimized antimicrobial peptide, which was named Ap920-WI (Figure S3).

**Figure 3:**
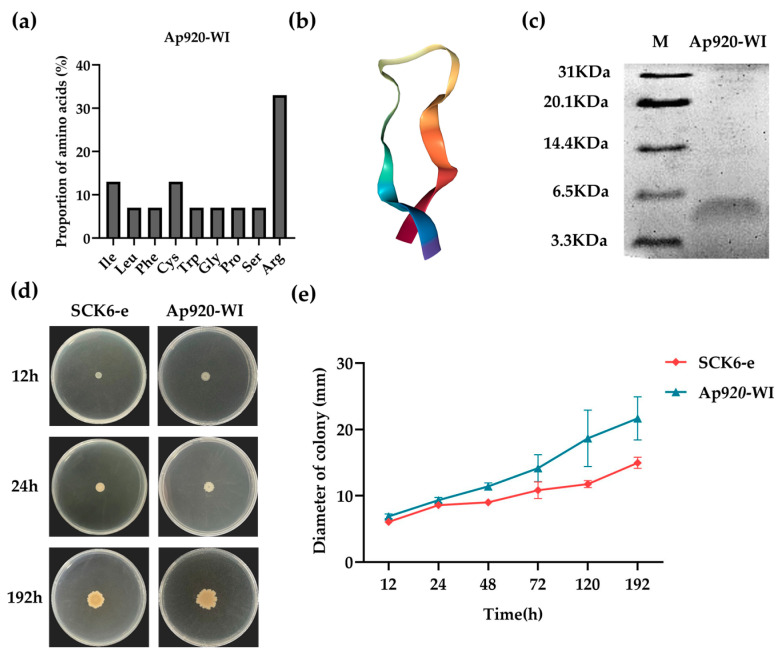
Bioinformatics analysis of Ap920-WI, Tricine-SDS-PAGE electrophoresis, and phenotype observation of transgenic *B. subtilis* bacteria. (**a**) The amino acid composition of Ap920-WI was analyzed according to the prediction results of APD3, and GraphPad Prism 9 was used for data visualization; (**b**) according to C-I-TASSER of the Zhang Lab, the 3D structure was predicted, and the NGL viewer 2.0.0 (http://nglviewer.org/ngl/, accessed on 28 February 2022) website was used to visualize the 3D structure. The pure antimicrobial peptide Ap920-WI sample was purified by Ni column, and the expression of the sample was detected by Tricine-SDS-PAGE and its size was verified. (**c**) Tricine-SDS-PAGE electrophoresis image: lane M is the pre-stained ultra-low molecular weight marker. The engineered *B. subtilis* bacteria containing the empty vector and the *Ap920-WI* gene were respectively spotted on LB (including Kan) plates and placed in a 37 °C incubator for incubation. The strains were regularly observed and photographed, and the growth diameter of each strain was recorded. (**d**) SCK6-e strain and Ap920-WI strain after culturing for 12 h, 24 h, and 192 h. (**e**) Data were analyzed and presented in a graph showing the mean of three independent experiments, with vertical bars representing the standard deviation (SD).

We conducted bioinformatics analysis and predicted the properties of Ap920-WI (Table S2). Results showed that compared to Ap920, Ap920-WI had only 15 amino acids, reducing its molecular weight to 1.9 kDa. Arginine, a hydrophobic amino acid, had the highest percentage in the amino acid composition, reaching 33% (Figure 3a). The hydrophobicity increased to 47%, and the PI value only decreased by 0.37. Although the number of positive charges decreased due to the reduction in the number of amino acids, the number of positive charges (+5) remained relatively high among antimicrobial peptides with the same number of amino acids. In the 3D structure (Figure 3b), Ap920-WI showed a similar β-sheet structure from the beginning to the end due to amino acid mutations and deletions.

### 2.4. Ap920-WI Has Antibacterial Activities with Lower MIC Values

To verify the antibacterial activity of Ap920-WI, we expressed the fusion peptide with His-tag and TEV restriction site in *B. subtilis* SCK6 using the same method as with Ap920. The purified peptide was verified using Tri-cine-SDS-PAGE, which showed that the Ap920-WI gene did not affect the host cells (Figure 3d). However, the band size on the protein gel was slightly larger than the predicted molecular weight (Figure 3c), likely due to factors such as the presence of His-tag and TEV cleavage site and the impact of voltage and current on peptide migration rate. These results indicate that Ap920-WI can be secreted extracellularly like Ap920 and can be purified by Ni column.

We also measured the minimum inhibitory concentration (MIC) value of Ap920-WI using the same method as with Ap920. The results showed that Ap920-WI had a MIC value ranging from about 35–100 μg/mL (Table 1). Compared to Ap920, Ap920-WI showed a higher MIC value for *C. fangii* but a lower MIC value for the other four pathogenic bacteria. We also tested the thermal stability (Figure S4a) and UV stability (Figure S4b) of Ap920 and Ap920-WI against *C. fangii, C. michiganesis,* and *X. oryzae* pv. *oryzicola*, respectively. After exposure to high temperatures and UV radiation, the antibacterial activity of Ap920 and Ap920-WI remained intact, with no significant changes in their effectiveness at 4 °C. In summary, these results indicate that Ap920-WI has stronger antibacterial activity compared to Ap920 and has the same thermal and UV stabilities.

### 2.5. Ap920-WI Has Antifungal Activities with Low Concentrations for 50% of Maximal Effect (EC_50_) Value

**Figure 4:**
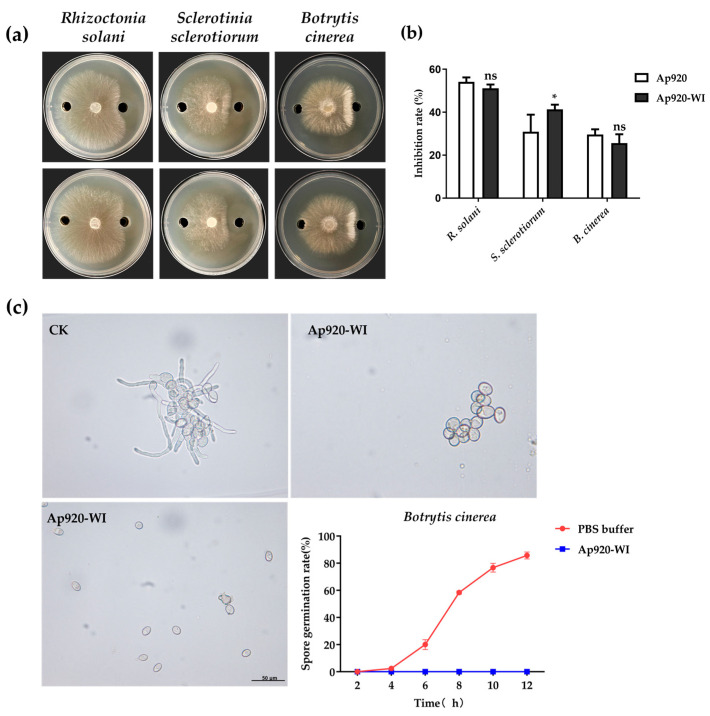
Antifungal activity of Ap920-WI. The PDA Oxford cup plate was inoculated with the indicator fungus, and the antifungal activity of the peptide was observed. (**a**) The antifungal activity of peptide from top to bottom: Ap920 to Ap920-WI; the control PBS buffer was added to the left, and the same amount of pure antimicrobial peptide was added to the right. (**b**) Data analysis of fungal inhibition rate comparing the antimicrobial activity with the control. *B. cinerea* was inoculated into the Potato Dextrose Broth (PDB) medium, and an equal volume of Ap920-WI was added to observe the inhibitory effect of the peptide on *B. cinerea*. (**c**) The inhibitory effect of Ap920-WI on *B. cinerea* at 12 h and the inhibition of growth kinetic curves. (CK) The control group was treated with PBS buffer. The data are the mean of three independent experiments; vertical bars represent SD (standard deviation). T-tests were carried out to evaluate the significance levels at * *p* < 0.05 and ns represents no significant difference, *p* > 0.05. Microscope magnification was 40 × 10.

The inhibitory ability of Ap920 and Ap920-WI against pathogenic fungi was explored using the Oxford cup method. It was observed that the same concentration of Ap920 and Ap920-WI could inhibit the mycelial growth of pathogenic fungi to varying degrees (Figure 4a,b). For *R. solani*, both Ap920 and Ap920-WI showed a significant inhibitory effect, forming a more obvious inhibitory arc with no significant difference in the inhibitory effect between the two. For *S. sclerotiorum*, Ap920-WI showed better inhibitory effect than Ap920, and the results were significantly different. However, for *B. cinerea*, the inhibitory effects of Ap920 and Ap920-WI were similar, and both resulted in the inhibition of mycelial growth, forming obvious mycelial “piling lines” near the Oxford cup hole containing antimicrobial peptides. To further explore the antifungal activity of Ap920-WI, the inhibition experiment of *B. cinerea* spores by microscope observation was carried out. The results showed that Ap920-WI completely inhibited the germination of *B. cinerea* conidia within 12 h and caused the conidia to rupture (Figure 4c), indicating that Ap920-WI may act on fungal hyphae and *B. cinerea* conidia, thereby inhibiting their germination.

The mycelial growth rate method was used to determine the concentrations for 50% of maximal effect (EC_50_) value against *R. solani*, *S. sclerotiorum*, and *B. cinerea* in order to better use Ap920-WI for the prevention and control of pathogenic fungi. The EC_50_ value of Ap920-WI was found to be around 25–65 μg/mL (Table 2), indicating its good application potential.

### 2.6. Inhibitory Effect of Ap920-WI on Mycelia Growth of Pathogenic Fungi

**Figure 5:**
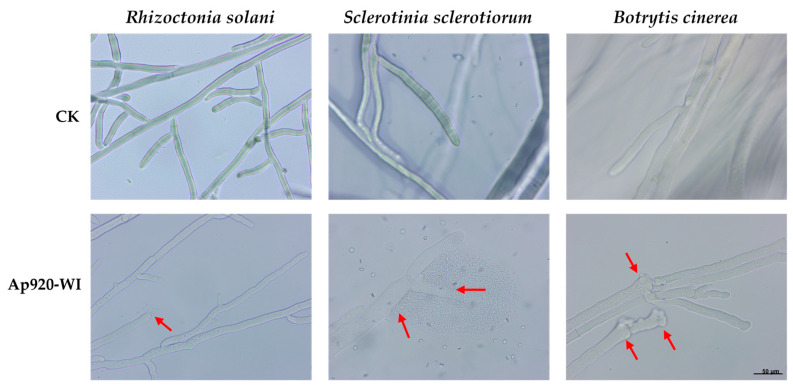
The effect of Ap920-WI on fungal hyphae was investigated by inoculating the indicator fungi onto a sterile glass slide with PDA medium. The inhibitory effect of the antimicrobial peptide Ap920-WI on mycelial growth was observed for pathogenic fungi including *R. solani*, *S. sclerotiorum*, and *B. cinerea*. The control was treated with PBS buffer. Red arrow means the hypha difference between CK and treatment The microscope magnification was 40 × 10.

To investigate the inhibitory effect of Ap920-WI on the mycelial growth of pathogenic fungi, *R. solani*, *S. sclerotiorum*, and *B. cinerea* were inoculated onto the center of Potato Dextrose Agar (PDA) medium on a sterile glass slide. PBS buffer solution and Ap920-WI were added dropwise to both sides as controls (see Figure S5). The inhibitory effect of the antimicrobial peptide Ap920-WI on mycelial growth was observed under a microscope. The results (Figure 5) showed that compared to the control, *R. solani* hyphae had abnormal morphology and hyphal tip rupture after treatment with antimicrobial peptide Ap920-WI, and *S. sclerotiorum* showed constriction at the hyphal septum and hyphal rupture. *B. cinerea* hyphae appeared with enlarged tips, branches, and abnormal branching. These phenomena indicated that the antimicrobial peptide Ap920-WI destroyed the cell membrane of the hyphae and formed membrane pores while inhibiting the growth of *R. solani* and *S. sclerotiorum* hyphae, leading to the leakage of hyphal contents. When inhibiting *B. cinerea* hyphae, it caused the mycelium branches to swell, making it difficult for the hyphae to grow.

### 2.7. Ap920-WI Has No Cytotoxicity to Mammalian Cells

**Figure 6:**
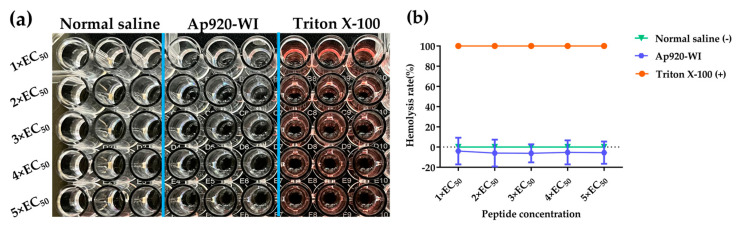
Ap920-WI has no cytotoxicity to mammalian sheep red blood cells. The experiment involved mixing Ap920-WI with 4% sheep red blood cells, culturing the mixture at 37 °C for 2 h, and then centrifuging it to obtain the supernatant in a 96-well cell culture plate. The OD_540_ value was measured using an ELISA (SPARK) plate reader. The negative control used was normal saline, while Triton X-100 was used as a positive control. The supernatant in the 96-well cell culture plate after treatment is shown in (**a**), while (**b**) shows the data analysis of the hemolysis rate. The data are the mean of three independent experiments; vertical bars represent SD (standard deviation).

To assess the biosafety of Ap920-WI, we conducted a hemolytic activity test using sheep blood cells. The results, as shown in Figure 6, demonstrated that the hemolysis rate of Ap920-WI was negative within the range of 1× EC_50_ value to 5× EC_50_ value, when compared with the negative and positive controls. This indicates that even at high concentrations, Ap920-WI does not cause hemolysis and may even have a protective effect on sheep blood cells. Therefore, it can be concluded that Ap920-WI is safe for mammalian cells.

### 2.8. Ap920-WI Can Inhibit the Infection of S. sclerotiorum on Rape Leaves

**Figure 7:**
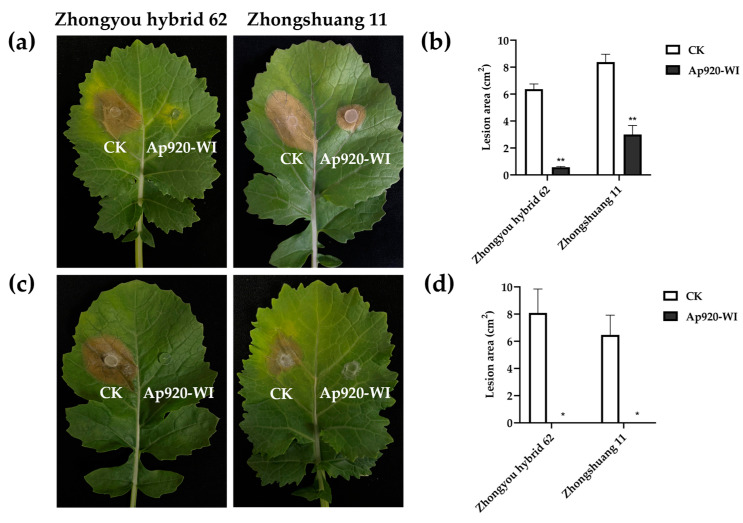
Resistance to *S. sclerotiorum* after treatment of rapeseed leaves with Ap920-WI. Rapeseed leaves were wounded using a 1 mL syringe needle and *S. sclerotiorum* cakes were placed on both wounded and non-wounded parts of the leaves. Ap920-WI peptide was applied to each cake for treatment. (**a**) The inhibition of *S. sclerotiorum* on rape leaves was evaluated using the micro-wound inoculation method, (**b**) the lesion areas were analyzed statistically, (**c**) The inhibition of *S. sclerotiorum* on rape leaves was also evaluated using the direct paste method. (**d**) The lesion areas were analyzed statistically. CK: PBS buffer. Data are the mean of three independent experiments; vertical bars represent SD (standard deviation). *T*-tests were carried out to evaluate the significance levels at * *p* < 0.05 and ** *p* < 0.01.

The study investigated the effect of applying antimicrobial peptide Ap920-WI on detached leaves of two rapeseed (*Brassica napus* L.) varieties, Zhongyouza 62 and Zhongshuang 11, using direct paste and micro-wound inoculation methods. The results revealed that in the micro-wound inoculation method, compared to the control, Ap920-WI inhibited *S. sclerotiorum* infection on detached leaves of Zhongyouza 62 and Zhongshuang 11 rapeseed by 82–96% and 56–74%, respectively. This indicates that Ap920-WI application can inhibit the growth of *S. sclerotiorum* in Zhongyouza 62 rapeseed leaves (Figure 7a,b). In the direct paste inoculation method, Ap920-WI inhibited *S. sclerotiorum* infection on detached leaves of both rapeseed varieties by 100%. Furthermore, although the *S. sclerotiorum* cake with Ap920-WI on detached leaves of Zhongshuang 11 rapeseed grew hyphae, it did not cause disease in rapeseed leaves (Figure 7c,d). This suggests that Ap920-WI inhibits the growth of *S. sclerotiorum* hyphae. These findings suggest that Ap920-WI has the potential to be an effective antimicrobial agent for controlling *S. sclerotiorum* infection in rapeseed.

## 3. Discussion

In recent years, there has been a growing interest in the development of novel synthetic antimicrobial peptides [29,30]. However, some of these peptides exhibit high cytotoxicity and hemolytic activity despite their remarkable antibacterial and bactericidal properties, due to their large amount of hydrophobic and positively charged amino acids [31]. Therefore, when designing synthetic antimicrobial peptides, it is important to balance these two factors. 

As bacterial antimicrobial resistance increases, the effectiveness of existing antibiotics is diminished [32]. Antimicrobial peptides with broad-spectrum antibacterial activity and low drug resistance have become a promising antibacterial strategy [33]. In this study, we obtained two antimicrobial peptides, Ap920 and Ap1182, screened from the genome library of *Arthrobacter* sp. H5 using the *B. subtilis* expression system. 

Some properties of antimicrobial peptides can be predicted by bioinformatics methods [34], and bioinformatics tools can be used to optimize the design of antimicrobial peptides to obtain antimicrobial peptides that are more suitable for applications [35,36]. After confirming Ap920 and Ap1182’s antibacterial activity, we used bioinformatics tools to predict and analyze their amino acid composition, 3D helical structure, and physical and chemical properties. We selected Ap920 as a template to design and synthesize a new antimicrobial peptide, Ap920-WI, which was successfully obtained. To design Ap920-WI, we first identified possible antibacterial activity sequence fragments in Ap920, and then optimized and transformed it into a 15-amino acid peptide by deleting certain amino acids and adding hydrophobic ones. During the amino acid mutation process, we substituted tryptophan (Trp, W) for arginine (Arg, R) to improve the bioavailability of the synthetic antimicrobial peptide and replaced half of the cystine (Cys, C) residues with the hydrophobic amino acid isoleucine (Ile, I) to increase the peptide’s hydrophobicity [37,38]. We used several bioinformatics websites to predict the antibacterial activity and hemolytic properties of the peptides, such as ClassAMP (http://www.bicnirrh.res.in/classamp/, accessed on 3 February 2022) [39] and AMPA (https://tcoffee.crg.eu/apps/ampa/do:ampa, accessed on 3 February 2022) [40] for predicting antibacterial activity and HemoNet (https://github.com/adibayaseen/HemoNet, accessed on 3 February 2022) [41] for predicting hemolytic properties. Although the predictions made by these websites are not always accurate, they can serve as reference indices for optimizing and transforming synthetic antimicrobial peptides. Although amino acid mutation and deletion can alter the properties of antimicrobial peptides, they may also affect their helical structure. In the case of Ap920-WI, the modified helix structure became like a β-sheet, whereas Ap920 was based on an α-helical structure. However, subsequent experiments confirmed that this change in helical structure did not affect the peptide’s antibacterial activity. 

SAMPs can be verified by chemical synthesis [42] and microbial heterologous expression [43]. To make synthetic antimicrobial peptides more suitable for agricultural biological control, we choose microbial heterologous expression to detect the antibacterial activity of SAMPs. After modifications, experiments were conducted on Ap920-WI to verify its anti-bacterial activity. First, the amino acid sequence of Ap920-WI was converted into a target gene sequence through codon optimization to meet the requirements of the expression system and restriction site. Then, the target gene sequence was connected to the target vector and finally transformed into *B. subtilis* SCK6 for verification of gene function. After the transformation of the Ap920-WI gene into *B. subtilis* SCK6, we observed the growth of the strain and found no damage to the host strain cells, such as autolysis and lysis. Moreover, the target peptide could also be purified using the same protein expression and purification conditions, indicating that Ap920-WI has no harm to *B. subtilis* and can be secreted extracellularly by *B. subtilis*. Subsequently, experiments were conducted to verify the antibacterial activity of Ap920-WI. It was found that Ap920-WI has antifungal activity against pathogenic fungi *R. solani* and *B. cinerea*, similar to Ap920, and both have heat and UV stabilities. Moreover, Ap920-WI has decreased MIC values against some pathogenic bacteria (*C. michiganensis, X. oryzae* pv. *Oryzicola, R. solanacearum,* and *X. oryzae* pv. *Oryzicola*), and it also has increased antifungal activity against the pathogenic fungus *S. sclerotiorum* with a lower MIC value. Due to the altered helical structure of the antimicrobial peptide Ap920-WI, more interest was focused on its antifungal activity. The EC_50_ results showed that Ap920-WI had lower EC_50_ values against *R. solani*, *S. sclerotiorum*, and *B. cinerea*. Ap920-WI completely inhibited the germination of *B. cinerea* conidia at twice the EC_50_ concentration. Microscopic observation revealed that Ap920-WI could cause abnormal mycelial growth and rupture of the outer membrane of the mycelium, resulting in the outflow of the content. In biological safety experiments, Ap920-WI showed absolute safety and a possible protective effect on mammalian cells. When applied to plants, Ap920-WI was found to inhibit the infection of rapeseed leaves by *S. sclerotiorum*.

As research on antimicrobial peptides deepens, more researchers are combining computer technology to develop new antimicrobial peptides with antibacterial activity [44]. Some researchers have used deep learning methods to build a model for mining potential new antimicrobial peptides and have synthesized 216 new antimicrobial peptides with antibacterial activity from the human intestinal microbiome [45]. Beneficial microbiomes and phyllosphere microbiomes have resulted in the excavation of more new bioactive potential and the discovery of more new antimicrobial peptides suitable for agricultural disease control, which have contributed to agricultural production.

## 4. Materials and Methods

### 4.1. Plant Material and Pathogen Culture

Rapeseed leaves were obtained from the Rapeseed Cultivation and Physiology Laboratory of Huazhong Agricultural University. The empty vector strain of *B. subtilis* SCK6 was transformed in our laboratory. *Arthrobacter* sp. H5 and pathogenic bacteria including *X. oryzae* pv. *Oryzicola*, *R. solanacearum*, *X. oryzae* pv. *Oryzae*, *C. michiganensis*, and *C. fangii* were cultured on lysogeny broth (LB) agar medium at 28 ± 1 °C, which was preserved in our laboratory. The pathogenic fungi *R. solani* AG-1, *B. cinerea* B05.10, and *S. sclerotiorum* 1980s used in the experiment were obtained from the Key Laboratory of Agricultural Microbiology, Huazhong Agricultural University, Wuhan, Hubei Province, China. The pathogenic fungi *R. solani* AG-1 was cultured on potato dextrose agar (PDA) medium and potato dextrose broth (PDB) medium at 28 ± 1 °C. The pathogenic fungi *B. cinerea* B05.10 and *S. sclerotiorum* 1980s were cultured on potato dextrose agar (PDA) medium and potato dextrose broth (PDB) medium at 20 ± 1 °C.

### 4.2. Arthrobacter H5 Genome Library Construction

Genomic DNA of *Arthrobacter* sp. H5 was extracted using the Cetyltrimethylammonium Bromide (CTAB) method with minor modifications [46], and the genomic DNA was fragmented by ultrasonic disruption (number of times: 30 times; time: 2 s; interval: 20 s; 25 HZ) and end-blunted with the Quick Blunting Kit. One end of the DNA fragment was made blunt, and the sticky end was created with a *Sma*I restriction enzyme, followed by endonuclease SibStar^®^*Pst*I (37 °C for 30 min, 80 °C for 10 min) single digestion of the genomic DNA. Finally, the *Arthrobacter* sp. H5 genomic DNA fragment was ligated into the pBE-S vector, transformed into *B. subtilis* SCK6, and cultivated overnight at 37 °C; a single colony was picked and shaken in 1 mL of Luria-Bertani (LB) medium (10 mg/L Kan) at 37 °C and 170 rpm/min for 5–6 h. The quality of the genomic library was evaluated using primers pBE-S-F (5′-GTTATTTCGAGTCTCTACGG-3′) and pBE-S-R (5′-TAACCAAGCCTATGCCTACA-3′). The library was stored at −80 °C.

### 4.3. Candidate Gene Screening and Confirmation

The transformants were spotted on LB agar medium plates (10 mg/L Kan) and cultured in a 37 °C incubator to observe the growth phenotype of the strains. After three rounds of screening, strains with abnormal growth phenotype were selected for the next experiment.

### 4.4. Extracellular Protein Extraction

Extracellular proteins were extracted by saturated ammonium sulfate precipitation. Engineered *B. subtilis* strains containing exogenous genes were activated on LB agar medium plates with kanamycin (10 mg/L) and cultured overnight at 37 °C. A single clone was selected and cultured in 100 mL of LB medium with Kanamycin (10 mg/L) and shaken at 170 rpm/min at 37 °C for 60–72 h using a Ruihua shaker in Wuhan, Hubei Province China. The culture was centrifuged at 9600× *g* for 20 min at 4 °C and the supernatant was collected. Saturated ammonium sulfate solution was slowly added dropwise while stirring the supernatant in an ice bath until it became turbid. The mixture was then refrigerated at 4 °C overnight, followed by centrifugation at 9600× *g* for 20 min at 4 °C. The precipitate was collected and the crude protein solution was dialyzed in PBS buffer (Na_2_HPO_4_, 0.3%; NaH_2_PO_4_, 0.6%, NaCl, 0.2%; (NH_4_)_2_SO_4_, 0.8%; pH 7.6) at 4 °C for 24–48 h. After dialysis, the crude protein solution was drawn into a 2 mL tube for later use. 

### 4.5. Antibacterial Activity Analysis

To determine the antibacterial activity of the extracted crude protein solution, the filter paper diffusion method was used [47]. Indicator bacteria (10^8^ CFU/mL) were added to semi-solid LB medium, which was then poured onto an LB agar medium plate. Sterilized 5 mm filter papers were placed on the medium plate, and 20 μL of crude protein solution was dropped onto each filter paper. The petri dishes were dried in an ultra-clean workbench and placed at the appropriate temperature for the specific indicator bacteria for 5–7 h. The diameter of the inhibition zone was observed and measured at any time.

### 4.6. Construction of the Fusion Gene with His-tag+TEV Restriction Enzyme Site

To obtain pure antimicrobial peptides, specific primers (Table S3) were designed to add His-tag and TEV restriction enzyme sites to the corresponding positions in the antimicrobial peptide coding sequence. This resulted in the construction of a fusion gene with His-tag+TEV restriction sites (Figure S6).

### 4.7. Extracellular Protein Purification and Tricine-SDS-PAGE

The fusion gene obtained in 4.6 was transformed into *B. subtilis* SCK6, and the extracellular protein was purified using Ni column (Kangwei Century Company, Beijing, China) affinity chromatography to obtain pure antimicrobial peptide. The crude protein extract obtained according to the method in 4.4 was combined with the filler in the Ni column and left overnight at 4 °C. After binding, impurity proteins were first eluted with PBS buffer containing 20 nM imidazole, followed by elution with PBS buffer containing 500 nM imidazole to obtain pure protein (peptide). The purified sample was mixed with 6× Tricine-SDS-PAGE loading buffer (Kangwei Century Company, Beijing, China)) at a 1:1 ratio, heated in a 100 °C water bath for 10 min, centrifuged at 9600× *g* at 4 °C for 3–4 min, and then separated using Tricine-SDS-PAGE gel (Kangwei Century Company, Beijing, China)).

### 4.8. Determination of MIC Value, Thermal Stability, and UV Stability

The MIC values of antimicrobial peptides were obtained by dilution to different concentrations. The concentration of the indicator bacteria was diluted to OD_600_ ≈ 0.02–0.05 and dropped into a 96-well cell culture plate; then different concentrations of antimicrobial peptides were added in equal amounts. After incubation at 28 °C for 24 h, the OD_600_ value was measured with an ELISA (SPARK) plate reader, and the minimum antimicrobial peptide concentration that could significantly inhibit the growth of the indicator bacteria was taken as the MIC value.

The antimicrobial peptide concentration was diluted to the MIC value of the indicator bacteria and then treated for 1 h at 50 °C, 60 °C, 70 °C, 80 °C, 90 °C, and 100 °C, as well as UV irradiation for 30 min, 60 min, 90 min, and 120 min. After treatment, equal volumes of indicator bacteria were mixed and incubated at 28 °C for 24 h and then the OD_600_ value was measured with an ELISA (SPARK) plate reader. The PBS buffer solution was used as a negative control, and the antimicrobial peptide treated at 4 °C was used as a positive control.

### 4.9. Bioinformatics Predictive Analysis and Optimization

To predict the physical and chemical properties, the 3D structure, and the most suitable N-terminus and C-terminus sequence peptides, various bioinformatics tools were utilized. The physical and chemical properties of antimicrobial peptides were predicted using APD3 (https://aps.unmc.edu/home, accessed on 18 February 2022) and Expasy (https://web.expasy.org/protscale/, accessed on 21 February 2022), respectively. The Zhang Lab (https://zhanggroup.org/, accessed on 28 February 2022) was used to predict the 3D structure. The BLAST analysis of antimicrobial peptides was conducted using NCBI (https://www.ncbi.nlm.nih.gov/, accessed on 18 January 2022). AntiBP Serve (https://webs.iiitd.edu.in/raghava/antibp/submit.html, accessed on 19 February 2022) was used to predict the most suitable N-terminus and C-terminus sequence peptides. Lastly, CAMP_R4_ (http://www.camp.bicnirrh.res.in/, accessed on 1 February 2022) was used to predict the probability of each site’s amino acid becoming an antimicrobial peptide.

### 4.10. Determination of the Inhibitory Effect of Antimicrobial Peptides on Pathogenic Fungi

The antifungal activity of antimicrobial peptides Ap920 and Ap920-WI was evaluated using the Oxford cup method [48]. The Oxford cup was placed in the designated position of an empty petri dish, and 30 mL of PDA medium was poured into it. The fungal cake was placed in the middle of the Oxford cup plate, and 200 μL of pure antimicrobial peptides and control were added to the hole of the Oxford cup. The plate was then incubated at different temperatures (28 ± 1 °C, 20 ± 1 °C), and the diameter of mycelium growth was measured and observed over time.

To observe the growth of mycelium, PDA medium was poured onto a sterile glass slide, and the fungal cake was inoculated into the center of the medium. Holes were punched on both sides with a 5 mm hole puncher, and 20 μL of Ap920-WI and control were added dropwise into the holes. The slide was then placed in the corresponding temperature incubator (28 ± 1 °C, 20 ± 1 °C) and incubated for 24 h. The growth of mycelium was observed using a microscope, and pictures were taken for recording.

### 4.11. Determination of Ap920-WI’s Inhibitory Effect on Botrytis Cinerea and Calculation of Its EC_50_ Value

*B. cinerea* spores (6.25 × 10^6^ spores/mL) were mixed with Ap920-WI in equal volumes, shaken at 150 rpm/min at 28 °C, and observed under a microscope every 2 h to count the number of spores germinated in 100 randomly selected spores. The EC_50_ value of Ap920-WI against pathogenic fungi was determined using the mycelial growth rate method. Different concentrations of pure Ap920-WI and a control were added to semi-solid PDA medium and poured into PDA medium plates, and pathogenic fungi were inoculated into the middle of the medium. The plates were incubated at 28 ± 1 °C or 20 ± 1 °C for 24–48 h, and the growth of mycelium was observed and measured to calculate the toxicity regression equation and determine the EC_50_ value.

### 4.12. Determination of Hemolytic Activity

Sterile defibrinated sheep blood was centrifuged at 664× *g* at 4 °C for 5 min to collect sheep red blood cells, which were washed with normal saline until the supernatant was clear and transparent. A 4% sheep red blood cell suspension was prepared. Different concentrations of pure peptides were prepared using physiological saline, mixed with an equal volume of erythrocyte suspension, and incubated at 37 °C for 2 h. After incubation, the mixture was centrifuged at 664× *g* at 4 °C for 5 min, and the supernatant was transferred to a 96-well cell culture plate to measure the optical density at 540 nm in the well using an ELISA (SPARK) plate reader. A red blood cell suspension treated with 1% Triton X-100 was used as a positive control, and a red blood cell suspension treated with normal saline was used as a negative control. 

The hemolysis rate was calculated according to the following formula: hemolysis rate (%) = [(A_peptide 540_ − A_normal saline 540_)/(A_Triton 540_ − A_normal saline 540_)] × 100.

### 4.13. Determination of Ap920-WI’s Effect on the Infection of S. sclerotiorum on Rapeseed

*S. sclerotiorum* was inoculated on a new PDA medium plate and cultured in an incubator at 20 ± 1 °C for 48 h. Mycelium blocks were selected using a puncher and placed directly on rapeseed leaves. A 1 mL syringe with a needle was used to make a micro-wound on the rape leaf, and 20 μL of antimicrobial peptide and the control were added dropwise. After 2–3 days, the diameter of the lesion was measured and recorded by taking pictures.

## 5. Conclusions

The research findings suggest that Ap920-WI has promising potential as a broad-spectrum antibacterial agent that can be utilized in the biological control of agricultural plant diseases. The use of engineered *B. subtilis* bacteria containing the Ap920-WI gene in large-scale fermentation is expected to be safe for host cells. However, further investigation into the mechanism of action of Ap920-WI is necessary to enhance its effectiveness and broaden its applicability in the agricultural sector. Overall, the results of this study provide a foundation for further research on the use of Ap920-WI in sustainable agriculture practices.

## Figures and Tables

**Table 1:** Minimum inhibitory concentration (MIC) values of antimicrobial peptides against five pathogenic bacteria.

Strains of Bacterium	Minimum Inhibitory Concentration (MIC: μg/mL)		
	Ap920	Ap1182	Ap920-WI
*Clavibacter fangii*	52	57	83
*Clavibacter michiganesis*	84	88	78
*Xanthomonas oryzae* pv. *oryzicola*	137	132	66
*Rastonia solanacearum*	67	64	37
*Xanthomonas oryzae* pv. *oryzae*	128	115	100

Note: Data are the mean of three independent experiments.

**Table 2:** The concentration for 50% of maximal effect (EC_50_) value of Ap920-WI.

Strains of Fungus	Regression Equation	EC_50_ (μg/mL)	Correlation Coefficient (R-Value)
*Rhizoctonia solani*	Y = 0.3996 + 3.1572X	29	0.9958
*Sclerotinia sclerotiorum*	Y = 1.3984 + 2.0141X	44	0.9805
*Botrytis cinerea*	Y = 1.9214 + 1.8783X	61	0.9834

Note: Data are the mean of three independent experiments.

## Data Availability

Not applicable.

## Supplementary Materials

The supporting information can be downloaded at: https://www.mdpi.com/article/10.3390/ijms241310598/s1.
