# Supplementary material for: Isolation and Optimization of a Broad-Spectrum Synthetic Antimicrobial Peptide, Ap920-WI, from Arthrobacter sp. H5 for the Biological Control of Plant Diseases
Source: Int J Mol Sci. 2023 Jun 25;24(13):10598. doi: 10.3390/ijms241310598 (PMC10341639; doi:10.3390/ijms241310598)
Supplement: Supplementary file 1 [file ijms-24-10598-s001.zip › ijms-2419429-supplementary.pdf]

Supplementary Materials

Isolation and optimization of a broad-spectrum synthetic antimicrobial peptide Ap920-WI from *Arthrobacter* sp. H5 for the biological control of plant diseases

Li Zhao, Md. Samiul Islam, Pei Song, Li Zhu, and Wubei Dong*

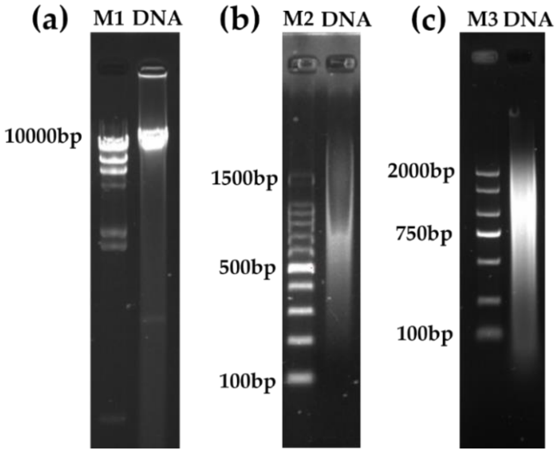

Figure S1. Genomic DNA quality assessment. (a) Total DNA was extracted using CTAB method, (b) Endonuclease *Pst*I monodigests Total DNA, and (c) Ultrasonic breaks Total DNA. M1: 1 Kb marker, M2: 100 bp marker, M3: DL2000 marker

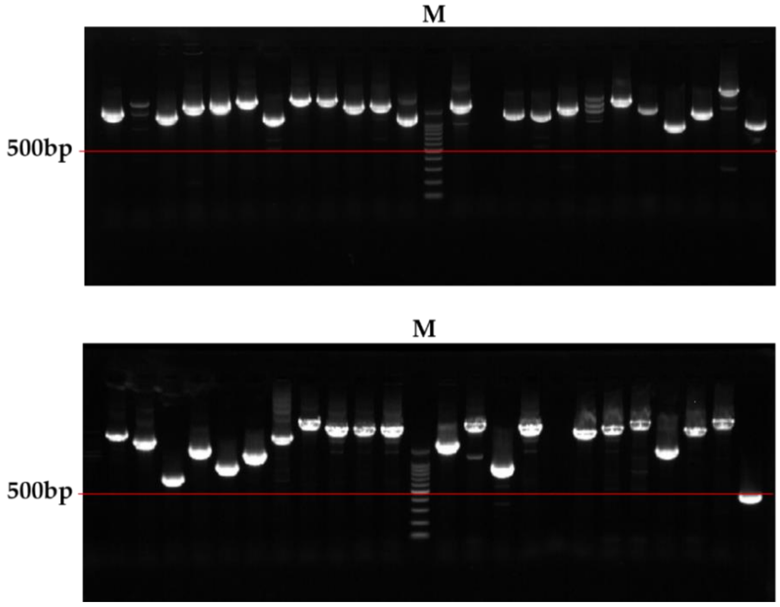

Figure S2. Agarose gel showing genomic DNA inserts. M: 100 bp marker

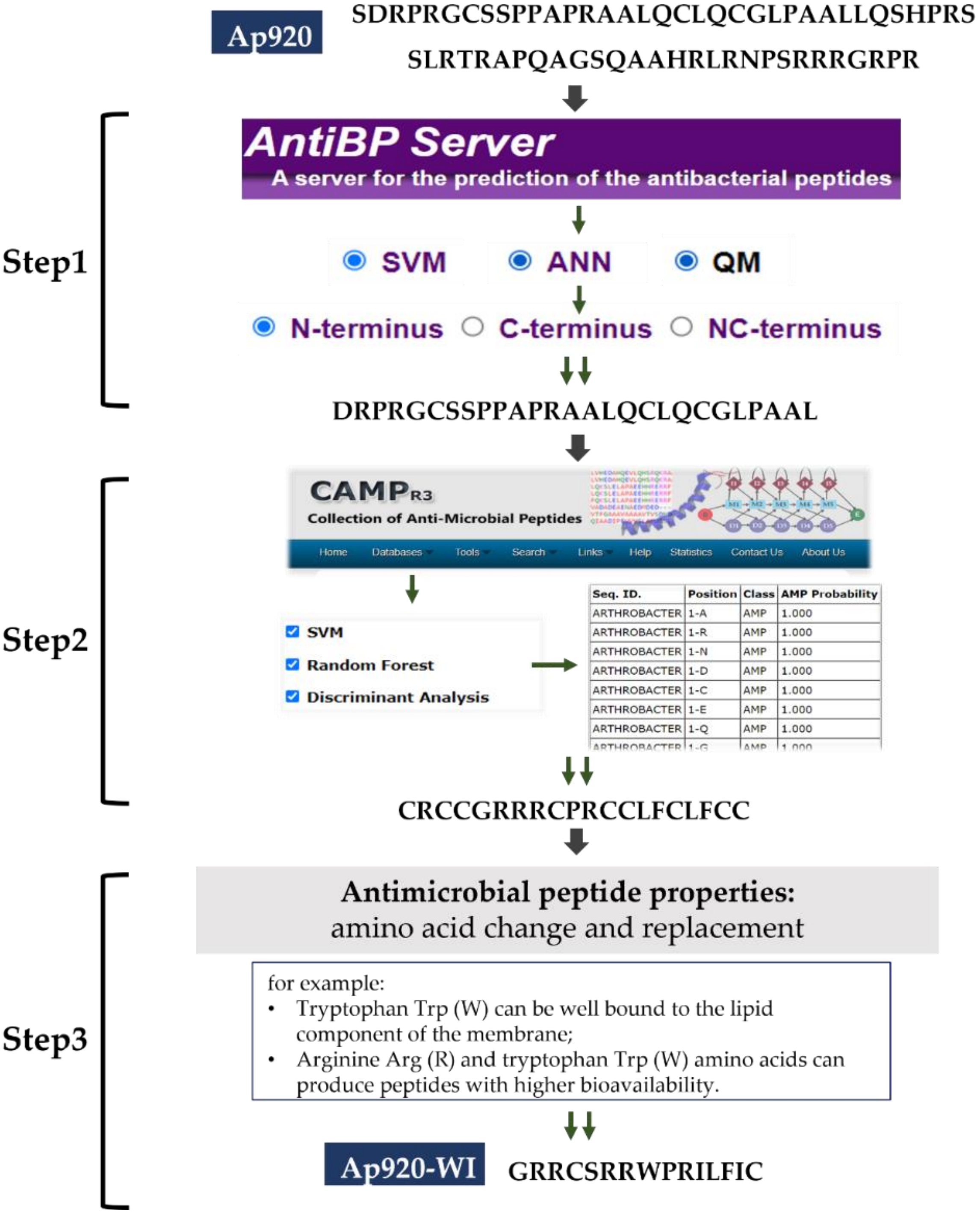

Figure S3. To transform the antimicrobial peptide Ap920 using bioinformatics methods and optimize its properties, the following steps were taken. In Step 1, the most suitable N-terminus and C-terminus sequence peptides for the three algorithms of SVM, ANN, and QM were predicted using the AntiBP Server. Step 2 involved predicting the likelihood of each amino acid site becoming an antimicrobial peptide using the three algorithms of SVM, RF, and DA on CAMPR3 (now CAMPR4). Finally, in Step 3, amino acid mutation and deletion were carried out based on the properties of the antimicrobial peptide, resulting in the final optimized antimicrobial peptide, Ap920-WI.

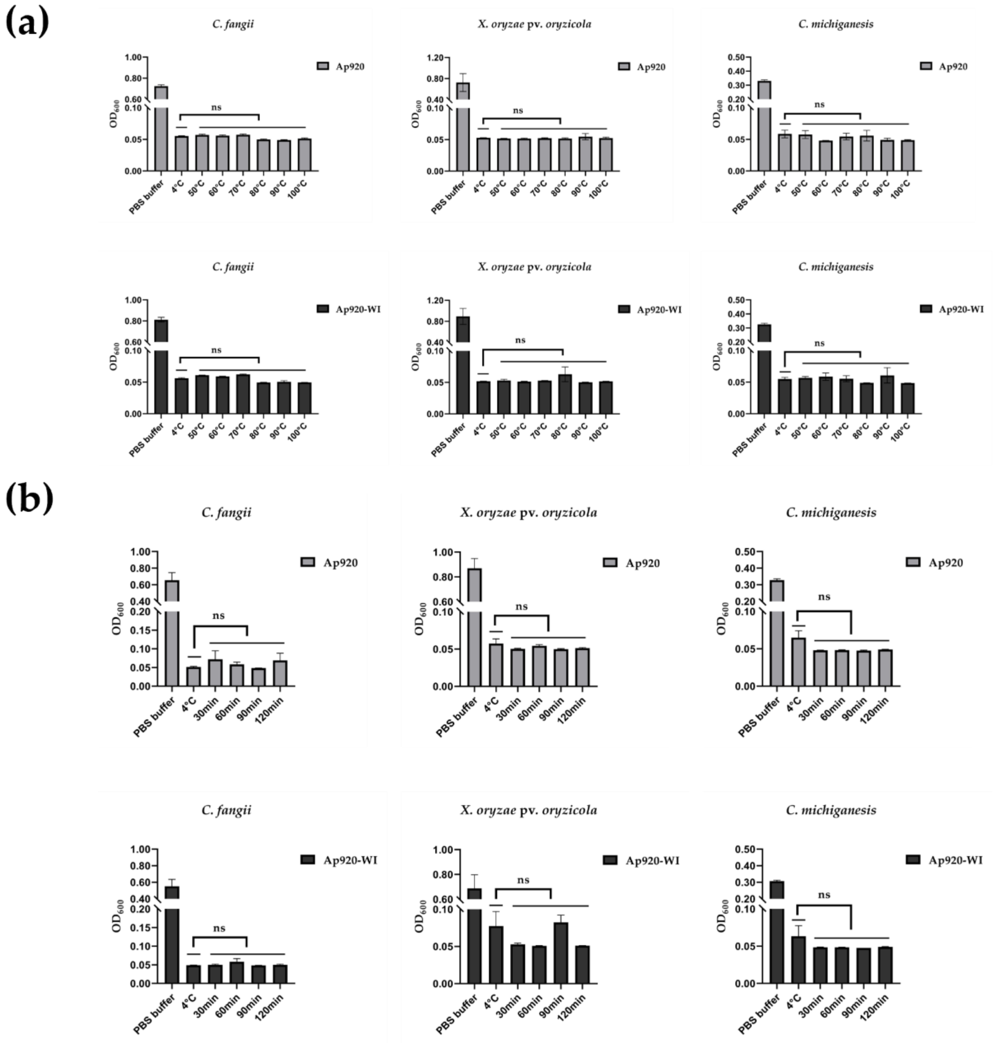

Figure S4. Ap920 and Ap920-WI thermal stability and UV stability results. Measure the OD_{600} value of antimicrobial peptides treated under different conditions mixed with indicator bacteria and incubated at 28°C for 24 hours and observe the change of OD_{600} value of antimicrobial peptides with MIC value and stored at 4°C. (a) Thermal stability diagram of Ap920 and Ap920-WI, (b) Thermal stability diagram of Ap920 and Ap920-WI. PBS buffer was used as a negative control, and antimicrobial peptides at 4°C were used as a positive control.

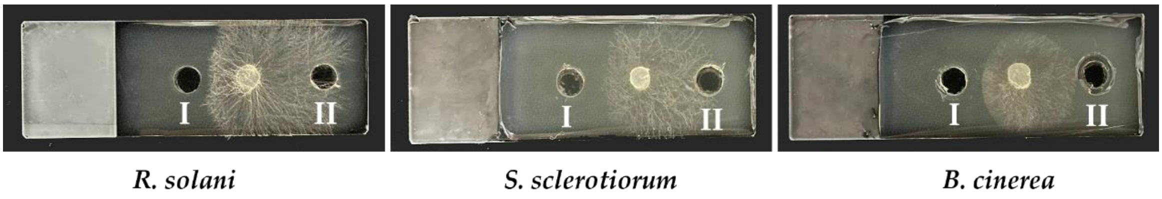

Figure S5. Microscopic observation of the inhibitory effect of Ap920-WI on the mycelial growth of pathogenic fungi. Firstly, PDA medium was poured onto a sterile glass slide. The fungal cake was then inoculated at the center of the medium. Holes were created on both sides of the fungal cake using a 5 mm hole puncher. Subsequently, 20 μl of Ap920-WI and the control were added drop by drop into the respective holes. The slide was prepared for microscopic observation to evaluate the impact of Ap920-WI on the growth of pathogenic fungi.

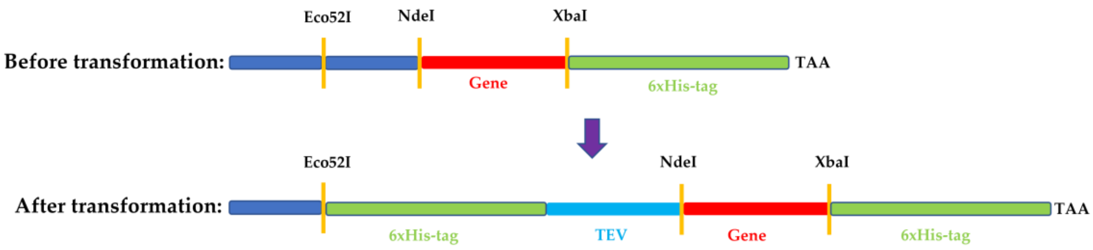

Figure S6. Construction of the His-tag+TEV restriction enzyme site fusion gene.

Table S1 Sequence information of candidate antimicrobial peptides

Antimicrobial peptides	AA	Amino acid sequence
Ap920	63	SDRPRGCSSPPAPRAALQCLQCGLPAALLQSHP RSSLRTRAPQAGSQAAHRLRNPSRRRGRPR
Ap1182	59	CLTTRGTLVADQRGCEVDCHYCRPYRRSAVICL GLPRRRLRSAGRTLAGRSLPPFQRPV
Ap920-WI	15	GRRCSRRWPRILFIC

Note, AA NO.: number of amino acids.

Table S2 Physicochemical properties of antimicrobial peptides

Physicochemical properties	Ap920	Ap1182	Ap920-WI
Hydrophobic Ratio	30%	37%	47%
Net Charge	+12.5	+9.25	+5
GRAVY	-0.98730158730159	-0.37457627118644	-0.3733333333333
The Wimley-White whole-residue hydrophobicity of the peptide	17.58	11.3	0
Molecular Weight	6833.845	6656.849	1919.362
molecular formula	$C_{282}H_{483}N_{111}O_{81}S_3$	$C_{283}H_{475}N_{99}O_{77}S_5$	$C_{84}H_{140}N_{31}O_{17}S_2$
molar extinction coefficient	187.5	3292.5	5675
Protein-binding Potential (Boman index) ）	3.45 kcal/mol	2.76 kcal/mol	3.62 kcal/mol
PI	12.20	11.05	11.83

Note, GRAVY: Grand Average of Hydropathy, PI: Isoelectric point.

Table S3 Primers and sequences purifying antimicrobial peptides

Primers	Sequences
TEV1-F	TACCCATCACCATCATCACCACGAAAACCTGTATTT TCAGTCCCATATGACAAGT
TEV1-R	CTAGACTTGTCATATGGGACTGAAAATACAGGTTTT CGTGGTGATGATGGTGATGGG
